# CircWHSC1 promotes ovarian cancer progression by regulating MUC1 and hTERT through sponging miR-145 and miR-1182

**DOI:** 10.1186/s13046-019-1437-z

**Published:** 2019-10-30

**Authors:** Zhi-Hong Zong, Yu-Ping Du, Xue Guan, Shuo Chen, Yang Zhao

**Affiliations:** 1 Department of Gynecologic Oncology Research Office, The Third Affiliated Hospital of Guangzhou Medical University, No.63 Duobao Road, Liwan District, Guangzhou, 510150 Guangdong China; 2 Department of Obstetrics and Gynecology, Key Laboratory for Major Obstetric Diseases of Guangdong Province, and Key Laboratory of Reproduction and Genetics of Guangdong Higher Education Institute in Guangdong Province, The Third Affiliated Hospital of Guangzhou Medical University, Guangzhou, 510150 China; 3 Department of Gynecology, the First Affiliated Hospital of China Medical University, Shenyang, 110001 China

**Keywords:** CircRNA, Ovarian cancer, Exosome, Tumorigenesis and progression

## Abstract

**Background:**

Circular RNAs are key regulators in human cancers, however, there is a lack of studies on circRNAs’ specific functions in ovarian cancer.

**Methods:**

Our study used qRT-PCR to detect the differentially expressed circRNAs between normal ovaries and ovarian cancer tissues. Cell function experiments were performed to verify the role of overexpression and silence of circWHSC1, including MTT assay, cell apoptosis assay, wound healing and Matrigel-coated Transwell assay. In vivo tumorigenesis model was constructed by subcutaneous injection in nude mice. Bioinformatics analysis predicted the possible binding sites of circWHSC1 with miRNAs, and confirmed with dual-luciferase reporter assay and RNA pull-down assay. The exosomes were extracted with ultracentrifugation. HE staining was also used to detect morphology of nude mice peritoneum.

**Results:**

We found that circWHSC1 was up-regulated in ovarian cancer tissues, and circWHSC1 expression was higher in moderate & poor differentiation ovarian cancer tissues than in well differentiation ovarian cancer tissues. Overexpression of circWHSC1 increased cell proliferation, migration and invasion, and inhibited cell apoptosis. Silence of circWHSC1 exerted the opposite effects. Additionally, circWHSC1 could sponge miR-145 and miR-1182 and up-regulate the expression of downstream targets MUC1 and hTERT. Exosomal circWHSC1 can be transferred to peritoneal mesothelial cells and promotes peritoneal dissemination.

**Conclusions:**

Our study demonstrates the highly expressed circWHSC1 in ovarian cancer promotes tumorigenesis by sponging miR-145 and miR-1182, and its exosome forms induce tumor metastasis through acting on peritoneal mesothelium.

## Background

As the most lethal gynecology malignancy worldwide, ovarian cancer accounts for 5% of female cancer deaths. In 2018, it is estimated that 22,240 new cases of ovarian cancer will be diagnosed in US [1, 2]. The anatomical features of the ovarian position deep in the abdominal cavity give the clinical features of its late diagnosis and low survival rates. Therefore, research on the molecular biological mechanism of ovarian cancer tumorigenesis and progression has received extensive attention.

CircRNAs are reported to be associated with some diseases and function as potential biomarkers for diagnosis. In particular, its “microRNA sponge” effect in tumorigenesis has been widely studied by researchers [3]. Due to the covalently closed loop structure, circRNAs have high tolerance to RNaseR exonuclease and remain more stable than its homologous linear transcript [4]. Recent studies showed that circRNAs are stably abundant in exosomes. Exosomal circRNAs were intact and enriched by at least 2-fold in exosomes compared to those in producer cells [5].

In this study, we have used gene chip technology to detect the presence of circular RNA in ovarian cancer in very few samples. We found that several circular RNAs including hsa_circ_0001387 (http://www.circbase.org/) are relatively highly expressed in ovarian cancer tissues and have not been reported in tumors. Circ_0001387 (from here on referred to as circWHSC1) is derived from exonic back-splicing of WHSC1 gene. We subsequently expanded the sample size and detected circWHSC1 expression and found that its expression level in ovarian cancer was higher than that in normal tissues. Therefore, we further explored underlying functions of circWHSC1 in ovarian cancer.

## Methods

### Ovarian cancer specimens

Seventy-nine epithelial ovarian carcinoma samples and 13 normal ovary samples were collected at the Department of Gynecology in First Affiliated Hospital of China Medical University (Shenyang, China). All the enrolled patients signed informed consents, and none of them had undergone chemotherapy or radiotherapy prior to surgery. Our study was approved by China Medical University Ethics Committee.

### Cell culture and transfection

Human ovarian carcinoma cell lines CAOV3 and OVCAR3 were cultured in RPMI 1640 (HyClone, USA) supplemented with 100 U/mL of penicillin/streptomycin and 10% fetal bovine serum. The incubator was set at 37 °C with 5% CO2. Lipofectamine3000 was used to perform transfection according to manufacturer’s instructions. The circWHSC1 lower-expressed cell line CAOV3 was stably transfected with pHBLV-CMV-circWHSC1 plasmid (Hanbio Biotech, Shanghai, China), while circWHSC1 higher-expressed cell line OVCAR3 was transfected with sh-circWHSC1-GFP targeting to destroy its circular structure. After transfection, both circWHSC1 overexpressed and down-regulated cell lines were further selected with 2 μg/mL puromycin. The sequences of circWHSC1 and shRNA can be found in Additional file 1: Table S3.

### MTT assay

The circWHSC1 overexpressed and down-regulated cells with control group were counted and seeded in 96-well plates. 0 h, 24 h, 48 h and 72 h after seeding cells, 20 μL of 5 mg/mL MTT solution (Solarbio, China) was added in each well, and incubated for 3 h. After discarding the supernatant, 150 μL of DMSO was added to dissolve formazan. Microplate spectrophotometer was used to detect the absorbance at 490 nm.

### Cell apoptosis assay

The cells were collected at 1500 r for 5 min and washed with PBS. 5 μL of 7AAD and PE-labeled Annexin V (BD Biosciences) were used to label apoptotic cells for each sample. The cells apoptosis rate was determined with flow cytometry.

### Wound healing assay

200-μL pipette tip was used to scratch wounds when cell confluence reached 80%. After washing with PBS, the cell culture medium was replaced with 20 μg/ml of mitomycin C in FBS-free medium, in order to weaken the interference effects of cell proliferation. The healing wounds were photographed twice at 0 h and 48 h after scratching.

### Transwell assay

50,000 cells were seeded onto filters coated with Matrigel in each chamber. 200 μL of serum-free medium was added at the upper compartment and 600 μL of medium with 10% FBS was added at bottom. After incubation for 48 h, cells above the filter were wiped off with a cotton swab, while the invaded cells at bottom were fixed, then stained with crystal violet.

### RT-qPCR

Total RNA from cells and tissues were extracted with TRIzol reagent. 5 μg of the RNA was reverse transcribed to complementary DNA and amplified using SYBR Premix Ex Taq™ II kit (Takara, Shiga, Japan). The comparative expression level of circWHSC1was compared with 18S with 2-ΔΔCt method.

### Western blotting

Total protein was extracted with RIPA buffer and boiled for degeneration. 30 μg of the protein were loaded into 7.5% SDS-PAGE. After being electrotransferred and blocking with 3% BSA, the membranes were incubated with primary antibodies. Rabbit against MUC1, CD63, E-cadherin, N-cadherin (1:1000; Proteintech, USA), hTERT (1:1000; Bioss, China), HSP70 (1:500; Boster, USA), TSG101 (1:1000; ABclonal, USA), β-actin (1:3000; Proteintech, USA) and mouse against CD9 (1:1000; Proteintech, USA) were used as primary antibodies. Anti-rabbit or anti-mouse IgG conjugated with HRP (1:5000; Abbkine, USA) was used as the secondary antibody. Enhanced chemiluminescence (Abbkine, USA) was used to display the bands.

### In vivo xenograft tumor model

4-week-old female BALB/c nude mice were subcutaneously or intraperitoneally injected with 1 × 10^7^ CAOV3 cells. To explore the role of circWHSC1-rich exosomes, the nude mice were divided into 2 groups after tumorigenicity. One group was intraperitoneally injected with 200 μg of exosomes isolated from circWHSC1-overexpressing CAOV3 cells, and the other group were injected with PBS as negative control. Both groups were injected every another 2 days. After 3 weeks, all the mice were sacrificed and tumor lesions were excised and photographed. The tumor volume (mm^3^) was calculated as length × width^2^ /2. All the mice were purchased from Vital River Laboratories (China), and kept in SPF environment at the Experimental Animal Center of China Medical University, which was approved by China Medical University Animal Care and Use Committee and complied with national animal experimental criteria.

### Dual-luciferase reporter assay

The binding sequences of miR-1182 and miR-145 on circWHSC1were cloned into pSI-check Dual-luciferase vectors (Hanbio Biotechnology, China). HEK-293 T cells were co-transfected with miR-1182 and miR-145 mimics and the wild type or mutated dual luciferase plamid. The cells were harvested at 48 h after transfection, and the luciferase activities were measured with Dual-Luciferase Reporter Assay System (Promega, USA). The relative luciferase intensity was normalized to renilla luciferase activity.

### RNA pull-down assay

BeaverBeads™ Streptavidin (BEAVER, SuZhou, China) was used to capture the biotin-labeled circWHSC1 probe, the empty vector was used as a control. The biotinylated nucleic acid compound was incubated with protein lysates of ovarian cancer cells OVCAR3 and CAOV3 cells overnight at 4 °C on the shaker, to specifically capture circWHSC1 and the corresponding conjugate. After elution of the magnetic beads, the bound RNA was extracted with TRIzol reagent, and the expression of miR-1182 and miR-145 were detected by qRT-PCR. The sequences of the probe against circWHSC1 can be found in Additional file 1: Table S3.

### Isolation of exosomes from cell culture medium

The cells were cultured in Exo-Clear™ cell growth medium (SBI, USA) for 36 h before extracting the supernatant. The 0.22 μm Millipore Express® PES Membrane (Millex, USA) was used to filter dead cells in supernatant. After 30 min of concentration at 10,000×g, the supernatant was ultracentrifugated at 100,000×g for 70 min (Optima XPN-100 Ultracentrifuge, Beckman, USA), and the pellet containing exosomes was collected. The pellet was washed in PBS and then concentrated at 100,000×g for another 70 min.

### Transmission electron microscopy

After ultracentrifugation, 2 μl of exosome pellet was dripped onto a carbon-coated copper grid and dried at 37 °C for 30 min. 20 g/L phosphotungstic acid was used to stained exosomes for 3 min. The morphology of exosomes was photographed by H-7650 transmission electron microscope.

### Cell morphology monitor

Before treated with exosomes, HMrSV5 cells were starved in serum-free medium for 12 h. After 24 h of co-culture with exosomal circWHSC1, which was extracted from circWHSC1-overexpressing cells conditioned culture medium, HMrSV5 cells were photographed.

### HE staining

The peritoneum of nude mice were excised and fixed in paraformaldehyde. After embedding and paraffin sectioning, the slices were dewaxed with xylene, dehydrated with gradient alcohol, and then stained with hematoxylin and eosin. The slices were photographed with Olympus light microscope (× 20).

### Statistics

All the data were collected at least three independent experiments and presented as mean ± SD. Two-tailed Student’s t-test and *P* < 0.05 were used to analyze statistical significance in SPSS 18.0 software.

## Results

### Correlation of circWHSC1 expression with epithelial ovarian carcinoma

**Fig. 1:**
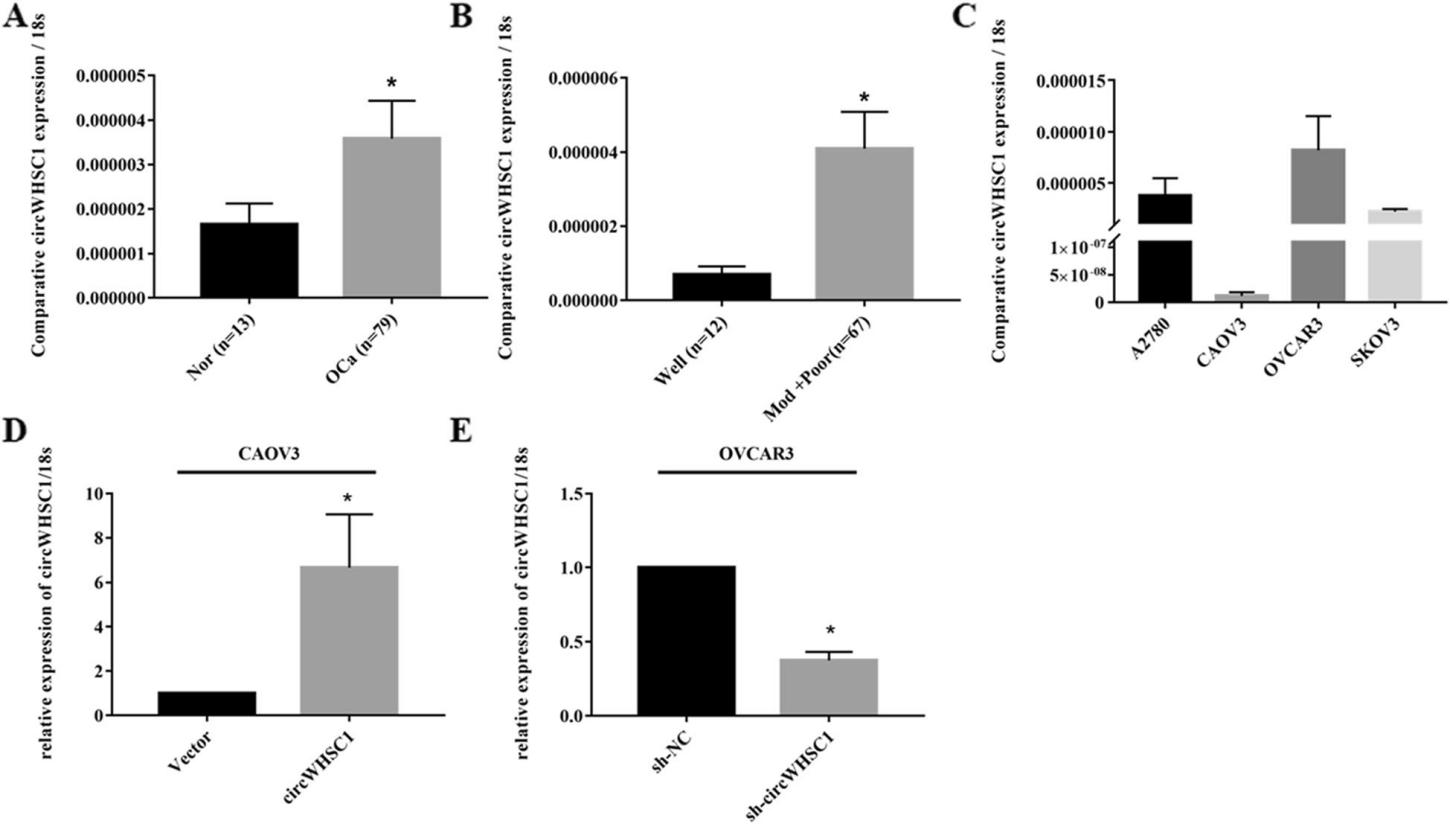
Correlation of circWHSC1 expression with the development and progression of epithelial ovarian carcinoma. The expression level of circWHSC1 in ovarian cancer tissues was higher than that in normal tissues (**a**), and was positively correlated with differentiation (**b**). The expression level of circWHSC1 was higher in cell line OVCAR3 compared with CAOV3 (**c**). QRT-PCR revealed that circWHSC1 was up-regulated in CAOV3, while it was down-regulated in OVCAR3 after transfection (**d**, **e**)

The expression level of circWHSC1 was determined with qRT-PCR. CircWHSC1 was significantly higher expressed in ovarian cancer tissues than that in normal ovaries (Fig. 1a, *p* < 0.05). Besides, circWHSC1 expression was positively correlated with differentiation (Fig. 1b, Well vs. Moderate and Poor, *p* < 0.05, details can be found in Additional file 1: Tables S1, S2).

### CircWHSC1 promotes cell viability, migration and invasion ability in ovarian cancer

**Fig. 2:**
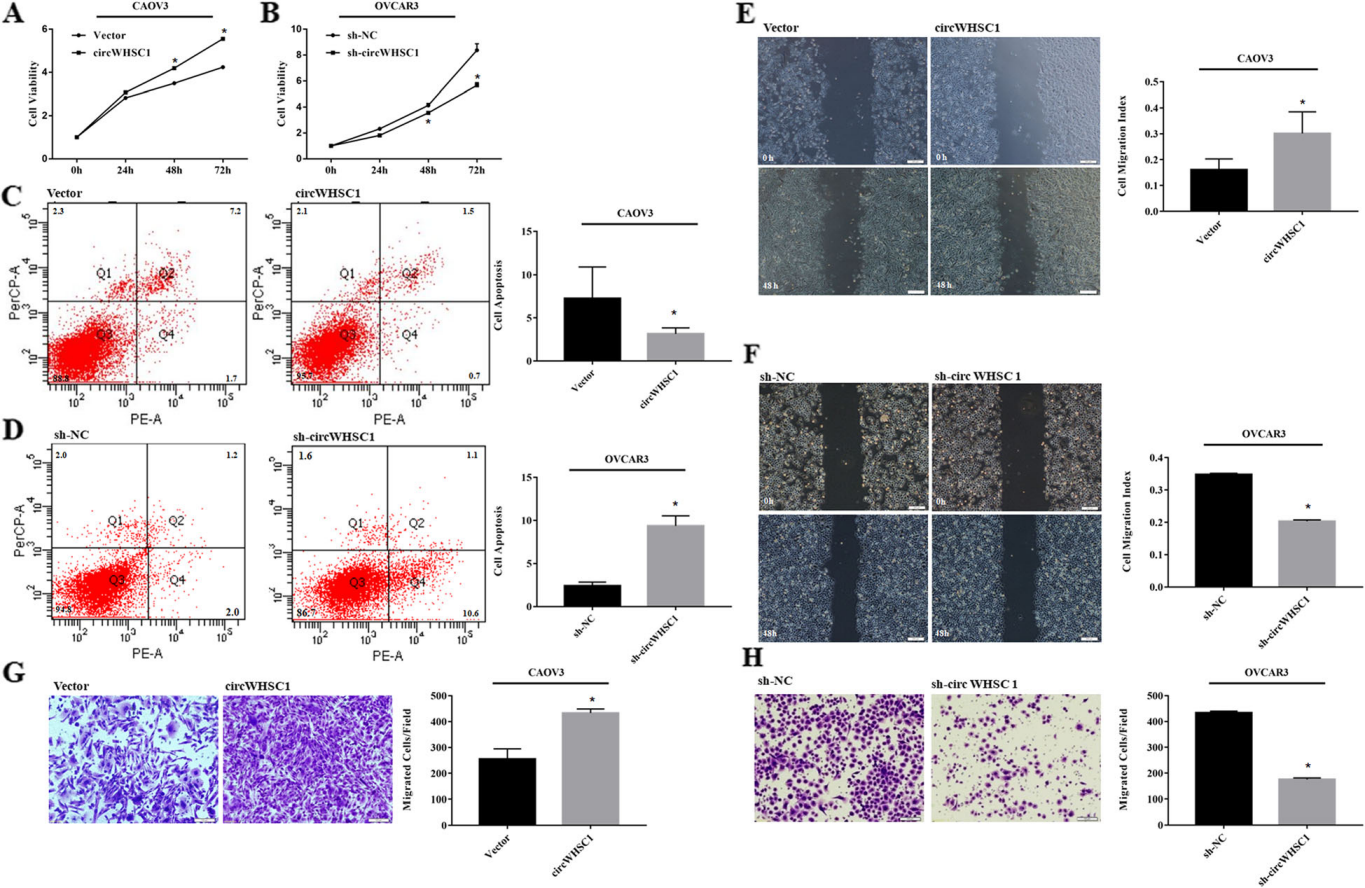
Effects of circWHSC1 on ovarian cancer cell viability, apoptosis, migration and invasion ability. Compared with the group transfected with control vector, overexpression of circWHSC1 in CAOV3 increased cell growth (**a**), reduced cell apoptosis (**c**), increased cell migration (**e**) and invasion rate (**g**). Down-regulation of circWHSC1 in OVCAR3 exhibited the opposite effects (**b**, **d**, **f**, **h**). The results are representative of three separate experiments; data are expressed as mean ± SD.* indicates *P* < 0.05

QRT-PCR indicated that expression level of circWHSC1 was highest in OVCAR3, and lowest in CAOV3 (Fig. 1c). After transfection of pHBLV-CMV-circWHSC1 plasmid, circWHSC1 was highly expressed in CAOV3 (Fig. 1d, *p* < 0.05). After sh-circWHSC1-GFP transfection, circWHSC1 was significantly down-regulated in OVCAR3 (Fig. 1e, *p* < 0.05). Compared with the group transfected with control vector, overexpression of circWHSC1 increased cell growth (Fig. 2a, *p* < 0.05), reduced cell apoptosis (Fig. 2c, *p* < 0.05), promoted cell migration (Fig. 2e, *p* < 0.05) and invasion rate (Fig. 2g, *p* < 0.05). Down-regulation of circWHSC1 in OVCAR3 exhibited the opposite effects (Fig. 2b, d, f, h, *p* < 0.05).

### Overexpression of circWHSC1 promotes tumor growth in vivo

**Fig. 3:**
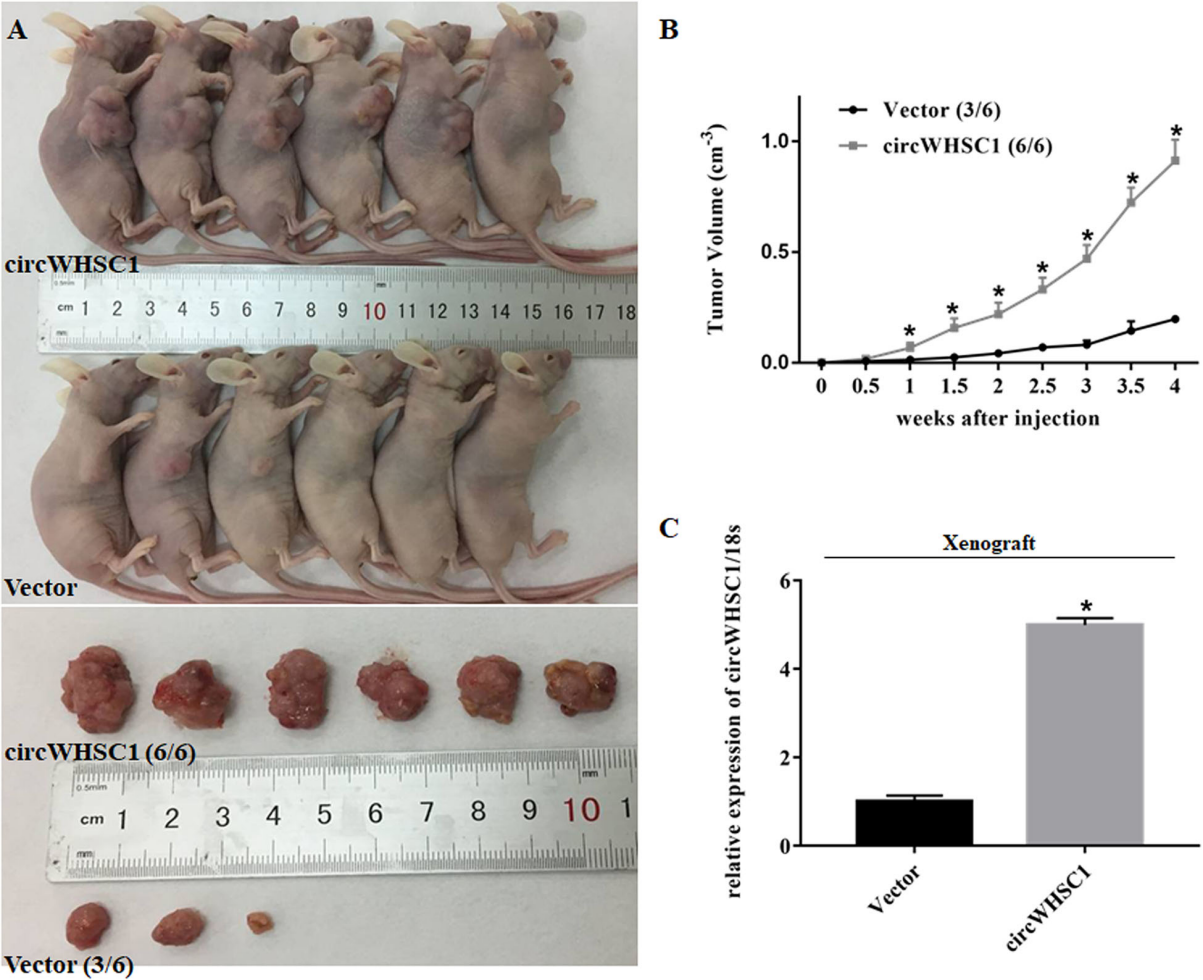
Effect of circWHSC1 on in vivo tumor growth. Compared with vector transfected group, subcutaneous injection of circWHSC1-overexpressed cells showed a more rapid rate of growth and larger tumor volume (**a**, **b**). The expression of circWHSC1 was significantly increased in subcutaneous xenograft of circWHSC1-overexpressed tumor cells (**c**). * indicates *p* < 0.05

Compared with vector transfected group, circWHSC1-overexpressed cells showed a more rapid rate of growth and the tumor volume was larger in nude mice (Fig. 3a, b, *p* < 0.05). And as shown in Fig. 3c, compared to control group, the expression of circWHSC1 was significantly increased in subcutaneous xenograft of circWHSC1-overexpressed tumor cells (*p* < 0.05).

### CircWHSC1 sponges miR-1182 and miR-145

**Fig. 4:**
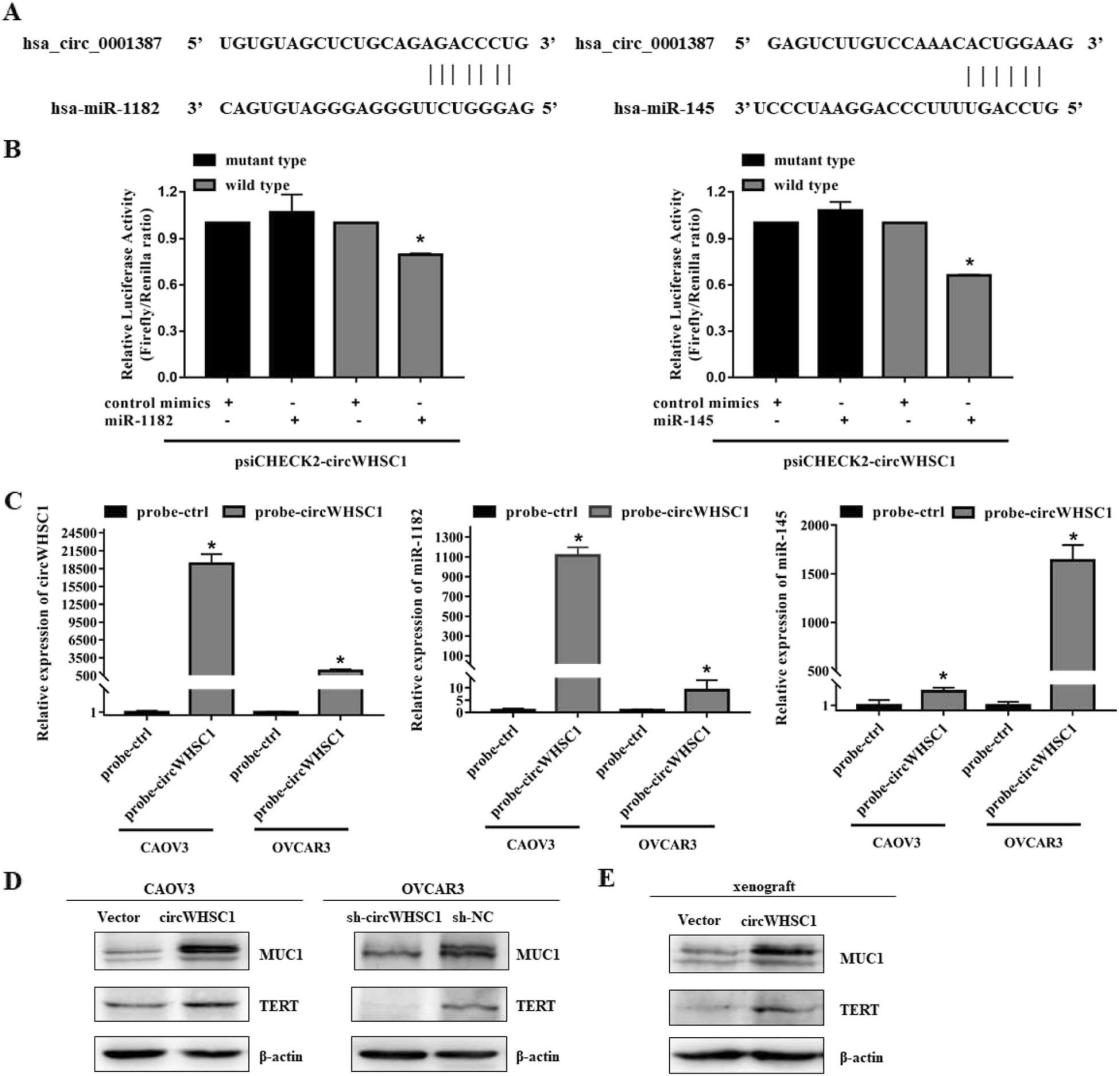
CircWHSC1 sponges miR-1182 and miR-145, and regulates expression of TERT and MUC1. The complementary sequences of complementary sequences with miR-1182 and miR-145 on circWHSC1 were predicted (**a**). Dual-luciferase reporter assay showed that both miR-1182 and miR-145 could significantly reduce the relative luciferase activity of the wild-type of circWHSC1 luciferase plamid compared with the mutant-type (**b**). RNA pull-down assay with a probe against circWHSC1 was used for qPCR analysis, which demonstrated an enrichment of circWHSC1 (left) and miR-1182 (medium) and miR-145 (right) in OVCAR3 and CAOV3 (**c**). Western blot showed that overexpression of circWHSC1 increased the expression levels of TERT and MUC1. Down-regulation of circWHSC1 yielded the opposite effects in OVCAR3 (**d**). TERT and MUC1 were also up-regulated in circWHSC1-overexpression xenograft (**e**). The results are representative of three separate experiments; data are expressed as mean ± SD.* indicates *P* < 0.05

Based on a circRNA/miRNA interaction prediction website (https://circinteractome.nia.nih.gov/), we predicted that there were complementary sequences with miR-1182 and miR-145 on circWHSC1 (Fig. 4a). Dual-luciferase reporter assay suggested that either miR-1182 or miR-145 could significantly reduce the relative luciferase activity of the wild-type of circWHSC1 luciferase plamid compared with the mutant-type (Fig. 4b, *p* < 0.05). RNA pull-down assay demonstrated that circWHSC1 binds directly to miR-1182 and miR-145. RNA from RNA pull-down assay with a probe against circWHSC1 was used for qPCR analysis, which demonstrated an enrichment of circWHSC1 and miR-1182 and miR-145 in OVCAR3 and CAOV3. (Fig. 4c, *p* < 0.05).

### CircWHSC1 regulates protein expression of TERT and MUC1

After overexpression of circWHSC1 in CAOV3, the expression levels of TERT and MUC1 were increased. Down-regulation of circWHSC1 yielded the opposite effects in OVCAR3 (Fig. 4d). TERT and MUC1 were also up-regulated in circWHSC1-overexpression xenograft (Fig. 4e).

### Exosomal circWHSC1 promotes peritoneal dissemination and regulates expression of MUC1 in peritoneum

**Fig. 5:**
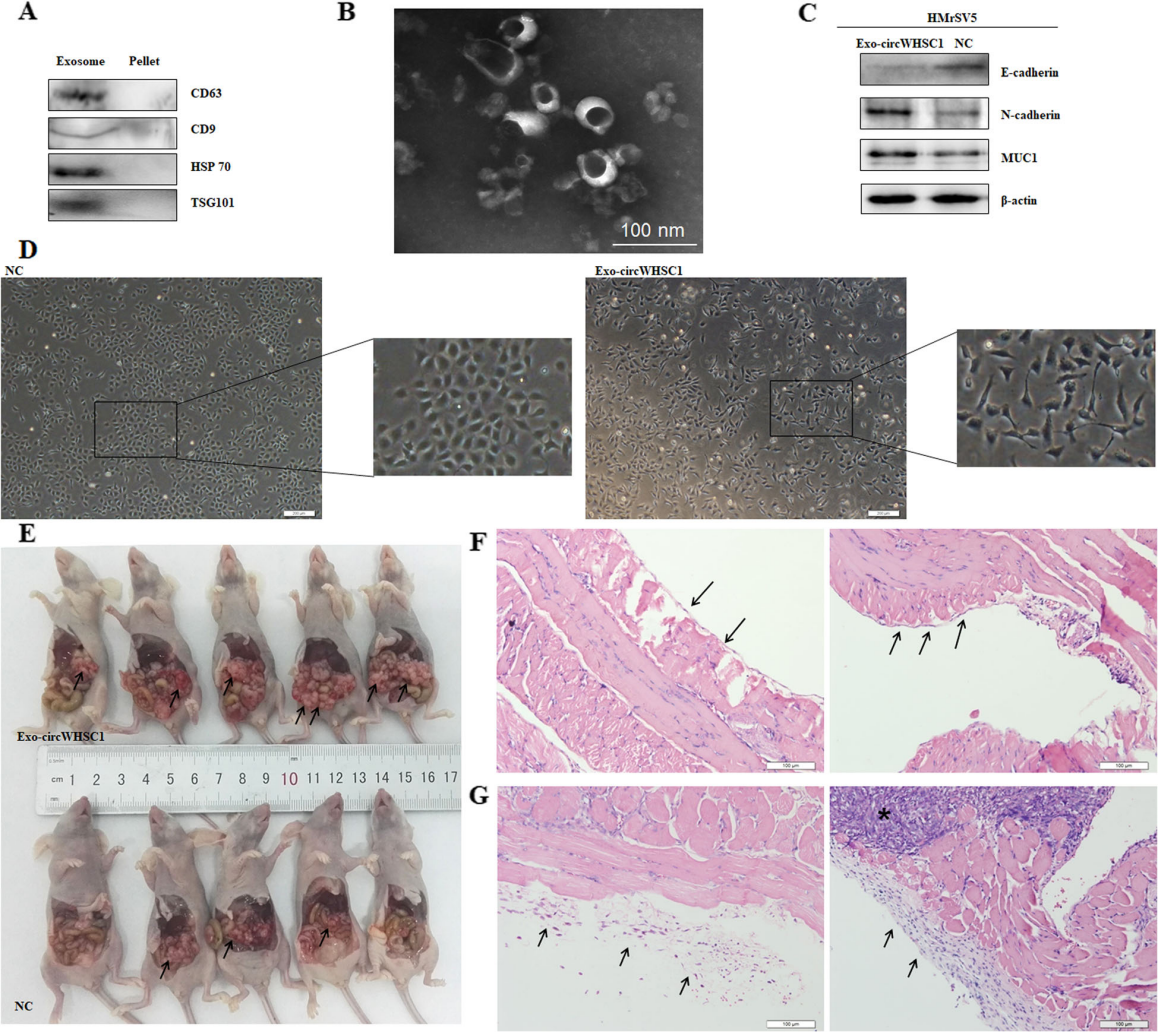
Exosomal circWHSC1 promotes peritoneal dissemination and regulates expression of MUC1 in peritoneum. Exosome specific markers, CD63, CD9, HSP70 and TSG101 were expressed in the extracted exosome pellets (**a**). Electron microscopy confirmed diameters of the extracted exosomes ranged from 10 to 100 nm and with cup shape appearance (**b**). The morphology of HMrSV5 cells incubated with exosomal circWHSC1 was converted into fibroblast-like (**c**). N-cadherin and MUC1 were up-regulated, while E-cadherin was down-regulated in exosome-treated HMrSV5 cells (**d**). After injection with circWHSC1 exosomes, the number of tumor nodules was significantly increased in abdominal cavity (**e**). HE staining showed that the peritoneum was covered with a monolayer of mesothelial cells with intact intercellular junctions from two different perspectives (**f**). After the treatment of exosomal circWHSC1, mesothelial cells arranged loosely, and the stromal layer was reactively thicken surrounding infiltration of tumor cells, and the left and right graphs represent two different perspectives (**g**)

Western blot showed exosome specific markers, such as CD63, CD9, HSP70 and TSG101 were expressed in the extracted exosome pellets (Fig. 5a). Electron microscopic indicated the diameters of the extracted exosomes ranged from 10 to 100 nm (Fig. 5b).

The morphology of HMrSV5 cells incubated with exosomal circWHSC1 was converted into fibroblast-like (Fig. 5c). After incubation of exosomal circWHSC1, N-cadherin and MUC1 were up-regulated, while E-cadherin was down-regulated in HMrSV5 cells (Fig. 5d). To explore the role of exosomal circWHSC1 in tumor peritoneal dissemination, nude mice were intraperitoneal injected with CAOV3 cells for tumorigenesis, and then injected with circWHSC1 exosomes or PBS as control group every 2 days. For the group treated with exosomes, the number of tumor nodules was significantly increased in abdominal cavity (Fig. 5e). HE staining showed that the peritoneum was covered with a monolayer of mesothelial cells with intact intercellular junctions (Fig. 5f). After the treatment of exosomal circWHSC1, mesothelial cells arranged loosely, and the stromal layer was reactively thicken surrounding infiltration of tumor cells (Fig. 5g).

## Discussion

In this study, we used circRNA microarray to screen for the differentially expressed circRNAs and discovered that hsa_circ_0001387 (http://www.circbase.org/) was highly expressed in ovarian cancer compared with normal ovaries. Furthermore, we used RT-qPCR to detect and quantify its expression level in 79 cases of epithelial ovarian carcinoma and 13 cases of normal ovarian tissues. The results showed that circWHSC1 expression level was significantly higher in ovarian carcinoma than normal ovaries, and was positively correlated with differentiation, which revealed that circWHSC1 might correlate with the development of ovarian carcinoma.

We performed cell function assays to verify circWHSC1’s functions in ovarian cancer. The specific shRNA targeting the splicing sites was used to destroy the circular structure of circWHSC1 in OVCAR3. Down-regulation of circWHSC1 suppressed cell proliferation, migration and invasion, and induced cell apoptosis. Overexpression of circWHSC1 in CAOV3 exerted the opposite effects. Compared with the control group, subcutaneous xenograft of circWHSC1-overexpressed tumor cells resulted in an increase in tumor formation ability and its volume in nude mice. These results indicated the highly expressed circWHSC1 plays an important role in tumorigenesis and development of ovarian cancer.

It has been reported that circRNA can adsorb microRNA and release its inhibitory effect on downstream targets [4]. Through competing endogenous RNA (ceRNA) network, circRNA regulates the expression of cancer-related genes and participates in tumorigenesis [6, 7]. Based on this theory, miR-145 and miR-1182 were found to contain complementary sequences with circWHSC1 according to bioinformatics prediction (https://circinteractome.nia.nih.gov/), which was further confirmed by dual-luciferase reporter assay and RNA pull-down assay in our study.

According to Hou et al. [8], up-regulation of miR-1182 suppressed cell proliferation, invasion ability, and decreased the expression of hTERT in ovarian cancer. TERT has been confirmed as a direct target of miR-1182 by luciferase assays. Similar effects could be found in gastric cancer [9] and bladder cancer [10]. In addition, miR-145 is down-regulated in ovarian cancer tissues and acts as a tumor suppressor. MiR-145 overexpression leads to the inhibition of colony formation, cell proliferation, cell growth viability and invasion, and the induction of cell apoptosis through directly targeting up-regulation of MUCl, which is also verified by luciferase report assays [11]. In our study, western blot assay showed that the expression levels of hTERT and MUC1 were increased in cells after up-regulation of circWHSC1, while down-regulation of circWHSC1 decreased the expression of both targets. Therefore, we concluded that circWHSC1 could affect the progression of ovarian cancer by sponging miR-1182 and miR-145, and regulated the expression of these two oncogenes.

Human telomerase reverse transcriptase (hTERT) acts as the major catalytic component of telomerase and regulates the extension of telomeres [12, 13]. TERT has been reported to be activated in cancer cell lines and plays an important role in growth, migration and invasion of cancer cells [14, 15]. In ovarian cancer cells, ectopic expression of TERT could induce epithelial–to-mesenchymal transition (EMT) by up-regulating of Slug [16, 17].

MUC1 (mucin 1) is a well-characterized member of membrane-bound mucins, modulates cell–to-cell and cell-extracellular matrix interactions and participates in cancer cell behaviors [18, 19]. As an oncogene, MUC1 is highly expressed in a variety of human adenocarcinomas, including pancreatic cancer [20], non-small cell lung cancer [21], gastric cancer [22] and ovarian cancer [23]. MUC1 coordinates invasive growth of tumor cells [24], triggers EMT process and participates in cancer metastasis, which is associated with poor prognosis [20–22]. In ovarian cancer, MUC1 is highly expressed compared to normal tissues and participates in cellular transformation and tumorigenicity [23].

In addition, Cho et al. [25] found that detecting the expression level of MUC1 in ascites can help distinguish between metastatic adenocarcinoma cells and reactive mesothelial cells, with a sensitivity of up to 99% and certain diagnostic value. Similar studies have reported that MUC1 immunoreactivity can be detected in ascites samples from patients with ovarian serous carcinoma and pancreatic cancer [26]. Moreover, compared with benign mesothelial cells, MUC1 was significantly higher in malignant mesothelioma [27]. Therefore, we wonder whether circWHSC1 can be secreted by ovarian cancer cells in the form of exosomes, taken up by peritoneal mesothelial cells, inducing up-regulation of MUC1 expression in mesothelial cells, and transformed into malignant mesothelial cells, which favors for peritoneal dissmination and tumor implantation.

Exosomes are small extracellular vesicles, function as vehicles of active protein and RNA, thus mediate cell-to-cell communications and regulates biological behavior of recipient cells [28–34]. We used PCR to confirm that circWHSC1 can be secreted into exosomes, furthermore, western blot showed exosome specific markers, such as CD63, CD9, HSP70 and TSG101 were expressed in the extracted exosome pellets and what’s more, electron microscopic indicated the diameters of the extracted exosomes ranged from 10 to 100 nm. The morphology of HMrSV5 cells treated with exo-circWHSC1 was fusiform-like, and the tight junctions between the cells were reduced, while the expression of E-cadherin was decreased, the expression of N-cadherin was increased, which indicated MMT process occurred in mesothelial cells. Similar with epithelial cells gain fibroblast-like phenotype and motility in EMT, MMT (mesothelial-to-mesenchymal transition) refers to mesothelial cells lining the peritoneal cavity convert into carcinoma-associated fibroblasts [35–37]. MMT dissociates intercellular junctions and increases cell adhesion and invasion through the peritoneum, which provides a suitable tumor environment for peritoneal implantation and dissemination [38, 39].

Further, we injected tumor cells into the abdominal cavity of nude mice, and the experimental group was given exo-circWHSC1 continuous stimulation. The results showed that the abdominal circumference of the nude mice increased, the abdominal cavity was transplanted, and HE staining showed that the peritoneal mesothelum lost its original tight structure between cells. In addition, western blotting showed that under exo-circWHSC1 stimulation, the peritoneal mesothelial cells expressed higher level of MUC1, which again verified the “microRNA sponge” effects of circWHSC1 in its receptor cells. Therefore, we put forward that circWHSC1 can be taken up by peritoneal mesothelial cells in the form of exosomes, and induces its EMT changes. Meanwhile, MUC1 is highly expressed, the adhesion of tumor cell to peritoneum and peritoneal dissemination are promoted.

## Conclusions

In summary, we found that circWHSC1, which is highly expressed in ovarian cancer, can adsorb miR-145 and miR-1182, up-regulates the expression of downstream targets MUC1 and hTERT, promotes cancer cell proliferation and invasion, also can be secreted into exosomes. In addition, peritoneal mesothelial cells can act as recipient cells and take up circWHSC1-rich exosomes. This process gives a favorable condition for tumor metastasis, which helps the tumor to spread in peritoneal cavity and promotes the progression of ovarian cancer. Our discovery enriches the research of the molecular biological mechanism of circRNA involved in the development of ovarian cancer, and provides novel ideas for new diagnostic and therapeutic strategies for clinical cancer therapy.

## Supplementary information

**Additional file 1.** The expression level and sequences of circWHSC1.

## Data Availability

The datasets used and/or analyzed during the present study are available from the corresponding author for reasonable request.

## Abbreviations

circRNA: Circular RNA
hTERT: Human telomerase reverse transcriptase
miRNA: MicroRNA
MUC1: Mucin 1
qRT–PCR: Real-time quantitative PCR
