# Supplementary material for: CircWHSC1 promotes ovarian cancer progression by regulating MUC1 and hTERT through sponging miR-145 and miR-1182
Source: J Exp Clin Cancer Res. 2019 Oct 30;38:437. doi: 10.1186/s13046-019-1437-z (PMC6822369; doi:10.1186/s13046-019-1437-z)
Supplement: Supplementary file 1 — Additional file 1. The expression level and sequences of circWHSC1. [file 13046_2019_1437_MOESM1_ESM.pdf]

Additional file 1: Table S1: circWHSC1 expression in normal ovary and ovarian carcinoma tissues

**Groups**	**N**	**circ WHSC1 expression / 18s**	***P* value**
Normal ovary	13	1.65E-06 ±4.73E-07	***0.0184***
Ovarian carcinoma	79	3.59E-06 ±8.55E-07	

Bold and Italics means P < 0.05.

Additional file 1: Table S2: Correlation of circWHSC1 expression with different clinicopathological features of ovarian carcinoma

**Clinicopathological features**	**N**	**circWHSC1 expression / 18s**	***P* value**
**The pathology types**			0.357
Serouscarcinoma	58	3.34E-06 ±6.19E-06	
The other pathology types	21	4.26E-06 ±1.07E-05	
**Age**			0.340
≤52	39	3.23E-06 ±5.97E-06	
> 52	40	3.94E-06 ±8.97E-06	
**FIGO stages**			0.330
I-II	28	4.18E-06 ±1.01E-05	
III-IV	51	3.26E-06 ±5.88E-06	
**Pathology classification**			***0.001***
Well	12	7.07E-07±7.58E-07	
Mod +Poor	67	4.10E-06±8.15E-06	

Bold and Italics means P < 0.05.

Additional file 1: Table S3: The sequence of circWHSC1 is

TGTTCTAAGAACGGAAGCATCTGGGCTGGATGGAATTTAGCATCAAGCAGAGTCCCC
TTTCTGTTCAGAGTGTTGTAAAGTGCATAAAGATGAAGCAGGCACCAGAAATCCTCG
GCAGTGCCAACGGGAAGACTCCGAGCTGCGAGGTGAACCGCGAGTGTTCTGTGTTCC
TCAGCAAAGCCCAGCTCTCCAGTAGCCTGCAGGAGGGGGTCATGCAGAAGTTTAACG
GCCACGACGCCCTGCCCTTTATTCCAGCCGACAAGCTGAAAGATCTTACTTCCCGGG
TGTTTAATGGAGAACCCGGCGCACACGATGCCAAACTGCGTTTTGAGTCCCAGGAAA
TGAAAGGGATTGGGACACCCCCTAACACTACCCCTATCAAAAATGGCTCTCCAGAAA
TTAAGCTGAAAATCACCAAAACATACATGAATGGGAAGCCTCTCTTTGAATCTTCCA
TTTGTGGTGACAGTGCTGCTGATGTGTCTCAGTCAGAAGAAAATGGACAAAAACCAG
AAAACAAGGCGAGAAGGAACAGGAAGAGGAGCATAAAATATGACTCCTTGCTGGAGC
AGGGCCTTGTCGAAGCAGCTCTTGTGTCTAAGATCTCAAGTCCTTCAGATAAAAAGA
TTCCAGCTAAGAAAGAGTCTTGTCCAAACACTGGAAGAGACAAAGACCACCTGTTGA
AATACAACGTTGGTGATTTGGTGTGGTCCAAAGTGTCGGGTTACCCTTGGTGGCCTT
GCATGGTTTCTGCAGATCCACTCCTTCACAGCTATACCAAACTTAAAGGTCAGAAAA
AGAGTGCACGCCAGTATCACGTACAGTTCTTTGGTGACGCCCCAGAAAGAGCTTGGA
TATTTGAGAAGAGCCTCGTAGCTTTTGAAGGAGAAGGACAGTTTGAAAAATTATGCC
AGGAAAGTGCCAAGCAGGCACCCACGAAAGCTGAGAAAATTAAGCTATTGAAACCAA
TTTCAGGGAAATTGAGGGCCCAGTGGGAAATGGGCATTGTTCAAGCAGAAGAAGCTG
CAAGCATGTCAGTGGAGGAGCGGAAAGCCAAGTTCACCTTTCTCTATGTGGGGGACC
AGCTTCATCTCAACCCTCAAGTAGCCAAGGAGGCTGGCATTGCTGCAGAGTCTTTGG
GAGAAATGGCAGAATCCTCAGGAGTCAGTGAAGAAGCTGCTGAAAACCCCAAGTCTG
TGAGAGAAGAGTGCATTCCCATGAAGAGAAGGCGGAGGGCCAAACTGTGTAGCTCTG
CAGAGACCCTGGAGAGTCACCCCGACATAGGGAAGAGTACTCCTCAAAAGACGGCAG
AGGCTGACCCCAGAAGAGGAGTAGGGTCTCCTCCTGGGAGGAAGAAGACCACAGTCT
CCATGCCACGAAGCAGGAAGGGAGATGCAGCATCCCAGTTTTTGGTCTTCTGTCAAA
AACACAGGGATGAGGTGGTAGCTGAGCACCCAGATGCTTCAGGTGAGGAGATTGAAG
AGCTGCTCAGGTCACAGTGGAGTCTGCTGAGTGAGAAGCAGAGAGCACGCTACAACA
CCAAGTTTGCCCTGGTGGCCCCTGTCCAGGCTGAAGAAGACTCTGGTAATGTAAATG
GGAAAAAAAGAAACCACACAAAGAGGATACAGGACCCTACAGAAGATGCTGAAGCTG
AGGACACACCCAGGAAAAGACTCAGGACGGACAAGCACAGTCTTCGGAAG

The sequences of shRNA targeting circWHSC1 are:

Top strand:

GATCCGAGTCTTCGGAAGTGTTCTAAGAACTTCAAGAGAGTTCTTAGAAC
ACTTCCGAAGACTTTTTTC

Bottom strand:

AATTGAAAAAAGTCTTCGGAAGTGTTCTAAGAACTCTCTTGAAGTTCTTA
GAACACTTCCGAAGACTCG

The sequences of probe against circWHSC1 are:

CAGAUGCUUCCGUUCUUAGAACACUUCCGAAGACUGUG
